# Supplementary material for: Effects of the Hypomethylating Agent Guadecitabine on Peripheral Blood Mononuclear Cell Methylomes and Immune Cell Populations in Small-Cell Lung Cancer Patients
Source: Pharmaceuticals (Basel). 2026 Mar 28;19(4):542. doi: 10.3390/ph19040542 (PMC13118959; doi:10.3390/ph19040542)
Supplement: Supplementary file 1 [file pharmaceuticals-19-00542-s001.zip › Figure S1.pptx]

## Slide 1
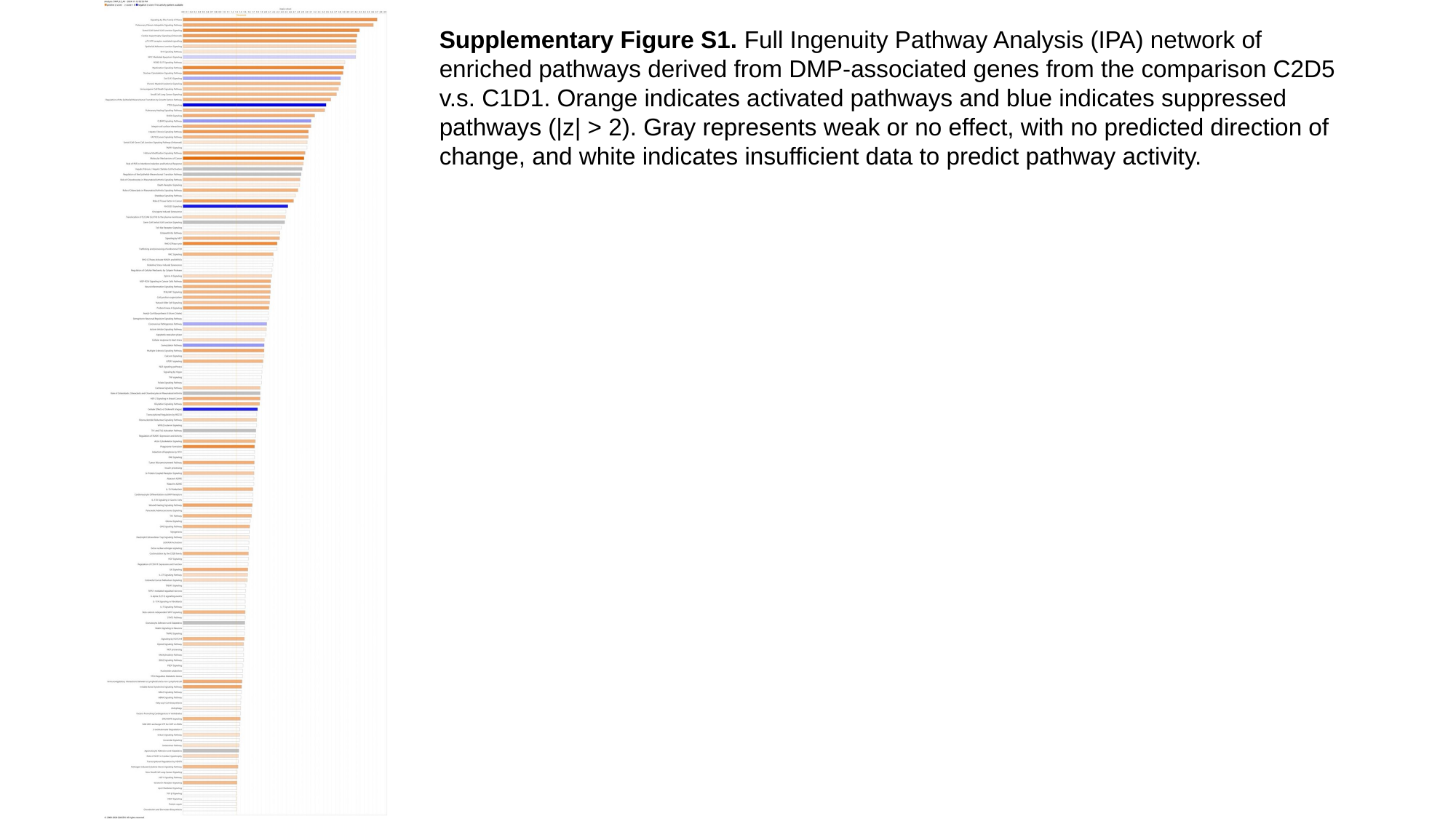

Supplementary Figure S1. Full Ingenuity Pathway Analysis (IPA) network of enriched pathways derived from DMP-associated genes from the comparison C2D5 v.s. C1D1. Orange indicates activated pathways and blue indicates suppressed pathways (|z| > 2). Gray represents weak or no effect, with no predicted direction of change, and white indicates insufficient data to predict pathway activity.
